# High-Q side-coupled semi-2D-photonic crystal cavity

**DOI:** 10.1038/srep26038

**Published:** 2016-05-19

**Authors:** Jianhao Zhang, Weixi Liu, Yaocheng Shi, Sailing He

**Affiliations:** 1 State Key Laboratory for Modern Optical Instrumentation, Centre for Optical and Electromagnetic Research, Zhejiang Provincial Key Laboratory for Sensing Technologies, East Building No. 5, Zijingang Campus, Zhejiang University, Hangzhou 310058, China; 2 Department of Electromagnetic Engineering, School of Electrical Engineering, Royal Institute of Technology (KTH), S-100 44 Stockholm, Sweden

## Abstract

High-Q semi-2D-photonic crystal cavities with a tapered edge and side-coupled bus waveguide are demonstrated. With a quadratic design, the unloaded cavity presents a theoretical ultrahigh quality factor up to 6.7 × 10^7^ for the condition that there are mere 34 holes in the propagated direction, which is pretty close to the 2D and 1D counterpart. Combined with a side-coupled bus waveguide, an all-pass-type cavity with a loaded quality factor (Q) of over 2.4 × 10^4^ and an extinction ratio over 10 dB are experimentally demonstrated. An experimental loaded Q up to 1.1 × 10^5^ are also achieved by tuning the coupling between the cavity and the bus waveguide, which is much larger than any reported surface-mode cavity. This cavity is quite suitable for sensors, filters and especially optomechanical devices thanks to the mechanical stability of the cavity and flexibility of the bus waveguide.

Photonic crystal (PhC) cavities, based on the emergence of bandgap, have been proven to be a viable alternative for high-performance resonators due to their high quality (Q) factor, small mode volume and FSR (free spectral range)-free operation. Various designs were introduced to improve the performance of PhC cavities, such as shifting the two adjacent right-and-left holes in a point-defect PhC cavity^1^, introducing a hetero-structure mechanism^2^ or introducing width modulation^3^,^4^ into the line-defect PhC waveguide, resulting in a Q value up to 2 × 10^7^. However, the 1D PhC cavity, which has a much smaller footprint than the 2D one, is also found capable of an equivalent or higher-level performance^4^,^5^,^6^. In addition to the comparable Q value and mode volume, a 1D PhC cavity, especially the nanobeam, has the ability to naturally couple to the feeding waveguide while maintaining a rather high Q value and high transmission^7^. In contrast, a 2D PhC cavity requires another line-defect PhC waveguide (that supports the resonant wavelength) or external microfiber to couple with the central region in order to interrogate its performance. In these 2D structures, there is a large mode mismatch between the input/output and line-defect PhC waveguide, which introduces a high coupling loss. Therefore, a 1D PhC cavity is more suitable for some efficiency-sensitive applications like sensing^8^,^9^, particle detecting and trapping^10^,^11^ though the 2D counterpart can also be implemented^12^,^13^.

Nevertheless, compared to the 1D PhC cavity, the 2D counterpart owns a larger area and can have many channels, and consequently has lower resistance to electrically-pumped lasers^14^ and better mechanical stability for applications requiring undercut, such as lasers^15^ and quantum dot-based devices^16^, since an undercut design can offer better mode confinement. A surface-mode PhC cavity, which has just half the footprint of the 2D counterpart and can be coupled to the bus waveguide^17^,^18^,^19^,^20^, have been proposed and experimentally demonstrated^21^,^22^. However, these surface-mode cavities have been formed by the Fabry-Perot effect, and both the intrinsic and the experimentally demonstrated Q values of the reported surface-mode cavities^17^,^18^,^19^,^20^ are much less than that of the 2D and 1D cavities. If this half-footprint cavity can be demonstrated to perform comparably to the 2D cavity, it may be pretty useful and adopted for further applications.

In this paper, we propose an ultrahigh-Q semi-2D-PhC cavity, which inherits the advantages of existing surface-mode PhC cavities while providing much better performance. Here three points are stressed. Firstly, the unloaded cavity is theoretically discovered to have excellent performance comparable to reported 2D and 1D photonic crystal cavities, while requiring just half the area of the 2D one. Secondly, the excitation of the cavity mode can be realized by a side-coupled waveguide, and a considerably high Q and extinction ratio with all-pass-type output spectrum can be achieved, better than any reported surface-mode cavity with nearly equal footprint. Thirdly, since the raw cavity is fixed to a large membrane while the movability can be endowed to the bus waveguide through an undercutting treatment, this device is also appropriate for optomechanical models, among which, for now, are mostly based on multi-mode ring resonators^23^,^24^, coupled waveguides^25^,^26^ and coupled nanobeam cavities^27^,^28^, in which the cavity also bears considerable movement. In addition, compare to the mushroom-like suspended ring or disk resonators with bus waveguide, this semi-2D-photonic crystal cavity presents same-level performance^29^,^30^,^31^ while providing better fabrication tolerance and repeatability for devices suspension. This high performance semi-2D cavity allows single-mode and cavity-static operations simultaneously. The identical-holes and period design of this cavity also promote further simplification of the fabrication process by multiple-beam interference lithography rather than Electron Beam lithography.

## Results

**Figure 1:**
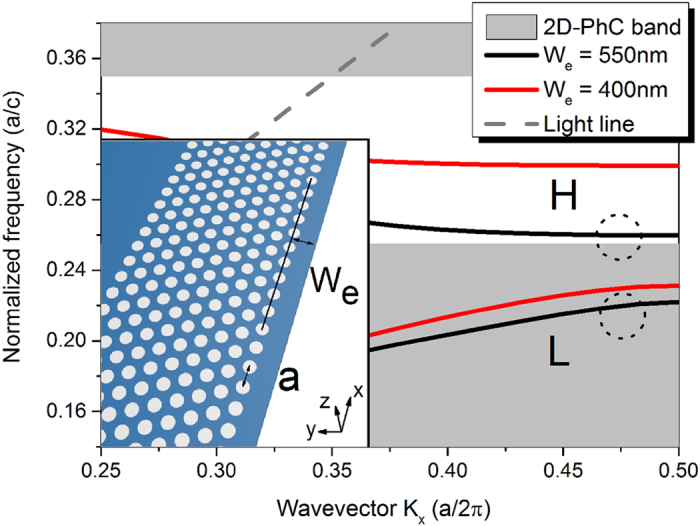
The band diagram of the Si-based semi-2D-PhC waveguide. Inset is the illustration of the waveguide, with period a = 420 nm. Si membrane thickness is fixed at 220 nm.

The dispersion diagram of the proposed semi-2D PhC waveguide (the structure is depicted in the inset), calculated by FDTD methods (Lumerical solutions, Inc.), is shown in Fig. 1. A silicon membrane with a 220 nm thickness and 3.46 refractive index is considered in our work. The PhC structure is formed by hexagonal-lattice PhC air holes and a redundant edge, where the lattice period and the distance between the silicon edge and the center of the adjacent holes are labeled as a and *W*_*e*_, respectively. The period *a* and radius of the air holes are fixed throughout the paper at 420 nm and 134 nm, respectively.

**Figure 2:**
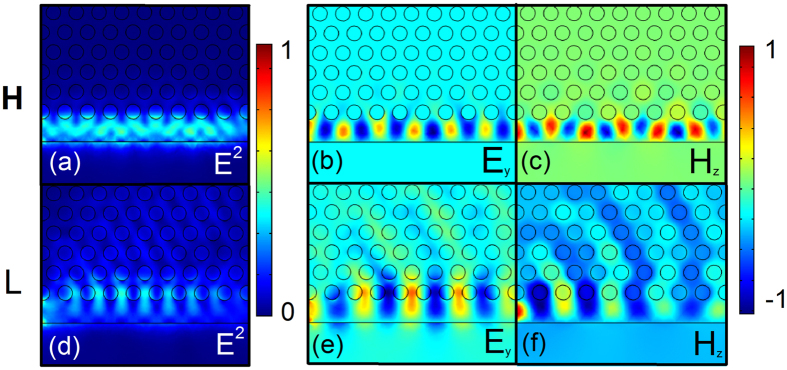
(**a,d**) is the power propagation of bandgap guided mode and index-guided mode, respectively. (**b,e**) is the corresponding y component of electric field and (**c,f**) the z component of magnetic field.

**Figure 3:**
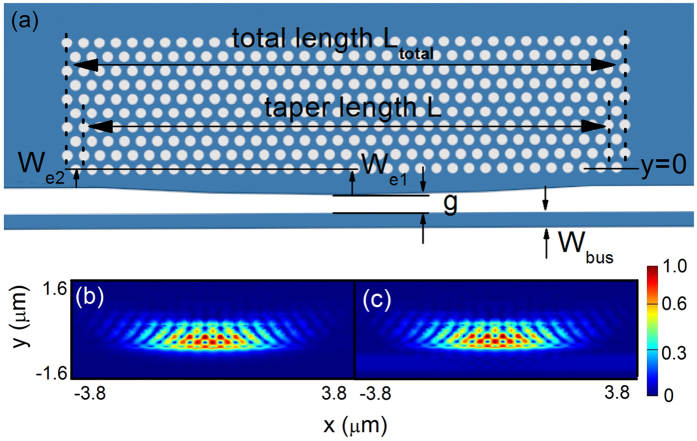
(**a**) Schematic of the side-coupled semi-2D-PhC cavity. (**b**) Time-averaged power distribution of the fundamental mode of the unloaded (without bus waveguide) cavity (**b**) and loaded cavity (**c**).

Figure 1 shows the dispersion curve for the proposed semi-2D PhC waveguide when *W*_*e*_ is chosen to be 400 nm and 550 nm (labeled by the red and blue curves, respectively). Transverse electric (TE) modes are considered in our work. The white region between the two greyed areas is the photonic bandgap (PBG) of the periodic 2D photonic crystal. Different from the conventional 2D-line defect-PhC waveguide, which totally depends on the (PBG) around, the mode we consider here is confined by PBG in one side and total internal reflection (TIR) for the other sides. In Fig. 1 we see that for each case there’re two dispersion curves with negative and positive dispersion, respectively. The lower curve is the index-guided mode that located below the PBG (labeled by “L”) and the higher one is the bandgap guided mode we discuss (labeled by “H”). The power propagation of these two mode is shown in Fig. 2(a,d), with normalized frequencies of 0.27 and 0.218, respectively. Compare to the PBG guided mode that greatly confined in the silicon edge, the index-guided mode have much energy leaked to the PhC side and poor propagation. The respective *y* component of electric field and *z* component of magnetic field is also shown in Fig. 2(b,c,e,f). Between these two dispersion curves, there is a large mode gap that can be used for the cavity design. Both dispersion curves shift to higher frequencies as *W*_*e*_ decreases (the dispersion curves for other *W*_*e*_ values have not been shown in Fig. 1). Specifically, the curve for the case of *W*_*e*_ = 550 nm is located near the center of the mode gap for the PhC with *W*_*e*_ = 400 nm. To create the mode gap effect [6], *W*_*e*_ is quadratically tapered from *W*_*e1*_ in the center toward both sides, i.e., *W*(*x*) = *W*_*e1*_ + *x*^2^(*W*_*e2*_ − *W*_*e1*_)/(*x*_max_)^2^ (*x* increases from 0 to *x*_max_). The proposed cavity structure with width modulation is depicted in Fig. 3(a). The PhC lattice contains n holes (n holes for the odd line and n + 1 for the even line) in the *x* direction and 10 lines in the *y* direction. *L* and *L*_*total*_ are the length of the taper segment and the total cavity, respectively.

Firstly, a 3D FDTD simulation is performed to obtain the performance of the unloaded cavity (without the side-coupled bus waveguide). The time-averaged power distribution of the fundamental mode of the unloaded cavity is shown in Fig. 3(b). We can find that most of the energy is confined in the silicon region, this is different from the previous surface-mode PhC cavity^17^,^18^,^19^ where most of the energy is confined in air holes and thin edges. This is also the reason why we name it a “semi-2D-PhC cavity” rather than a “surface” one.

**Figure 4:**
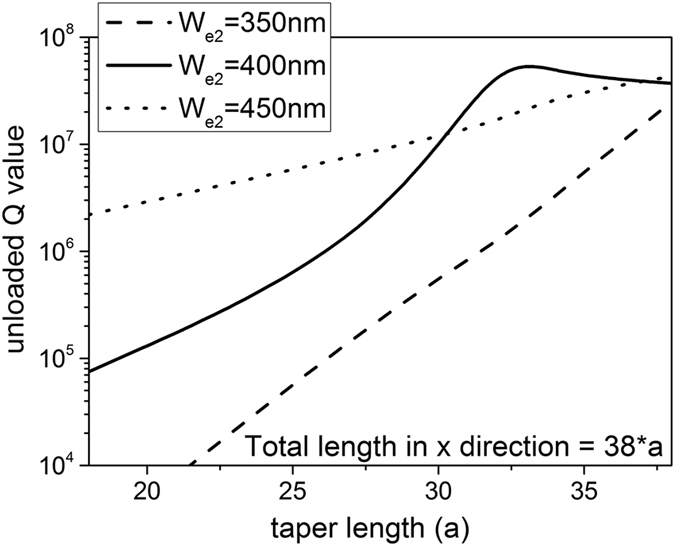
Simulated Q value by tuning the *W*_*e1*_ and taper length *L*.

The performance of the cavity is strongly dependent on the edge width *W*_*e2*_ and taper length *L*. We use a deterministic design method proposed in [6] to optimize our cavity. The taper region is defined with a quadratical modulation in order to get a Gaussian type confinement. Fig. 4 shows the Q values of the unloaded cavity with different *W*_*e2*_ and taper length *L* while the total number of holes is 38 in the *x* direction (e.g. *L*_*total*_ = 37*a) and *W*_*e1*_ is fixed to 550 nm. As shown in Fig. 4, the highest Q (>10^7^) can be achieved at *W*_*e2*_ = 400 nm, which is consistent with the analysis of the band diagram shown in Fig. 1. If the taper section is fixed (with fixed shape and length), there’s no doubt that a longer cavity would give a higher Q value. However, for a cavity with a fixed length, the Q value depends on the on the tapering shape, instead of the taper length. The highest Q value results from the cavity with a Gaussian-shape mode distribution by changing the taper length as shown in [6]. Based on this, the optimal parameters for the cavity are: *W*_*e1*_ = 550 nm, *W*_*e2*_ = 400 nm, *L* = 31*a and *L*_total_ = 33*a (32 holes and 34 holes, respectively, as we cut down the total holes to that of the taper plus two), resulting in a Q value of 6.7 × 10^7^ and a mode volume V of 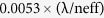
^3^ for the fundamental mode (1547 nm). If we fixed the cavity length and increase the taper length to a certain value larger than that of cavity, the Q value for case *W*_*e2*_ = 350 nm will be close to (but not larger than) that of *W*_*e2*_ = 400 nm since the taper edge is quite similar within the cavity length in these two cases. For the structure with *W*_*e2*_ = 450 nm, the Q value can be higher than that of case *W*_*e2*_ = 400 nm by increasing the taper length in the longer cavity. In our work, the total number of periods for the cavity in x direction is chosen to be 38 for a comparison with the existing 2D, 1D and surface-mode cavities, and also for easing the fabrication. The upper limit for the Q factor depends on the deviation from the designed mode and the Gaussian-shape mode distribution along the taper direction, which has also been discussed in [6].

Next, a bus waveguide with width *W*_*bus*_ is placed adjacent to the cavity with a gap *g* to excite the cavity mode. To enhance the matching between the PhC cavity mode and the bus waveguide mode, the waveguide width is chosen according to (2*π × n*_*eff*_*/λ*) = (*π*/a), where *n*_*eff*_ is the effective index of the bus waveguide. *W*_*bus*_ = 350 nm and *g* = 500 nm are selected in advance to achieve a relatively complete coupling that usually exhibits a moderate Q and high extinction ratio (ER) value. The power distribution of the excited fundamental mode is exhibited in Fig. 3(c). Compares to the unloaded cavity mode, this loaded mode has much coupled power to the bus wavguide, just as the bottom region of Fig. 3(c), though they share similar profile inside the cavity. Because of the perturbation of this bus waveguide, Q drastically decreases to about 10^4^ with an ER of about 15 dB. However, it should be noted that a higher Q value still can be achieved by slightly changing the mode matching and coupling strength through tuning the waveguide width and gap. When adjusting the waveguide width from 350 nm to 375 nm while keeping the gap at 500 nm, the Q value of the 1^st^, 2^nd^ and 3^rd^ modes can be raised to 3.2 × 10^4^, 9.8 × 10^4^ and 2 × 10^5^, respectively, while maintaining a considerable ER up to 10 dB for the 1st mode. In this situation, only the energy of the 1^st^ mode would almost be dropped into the PhC cavity completely.

**Figure 5:**
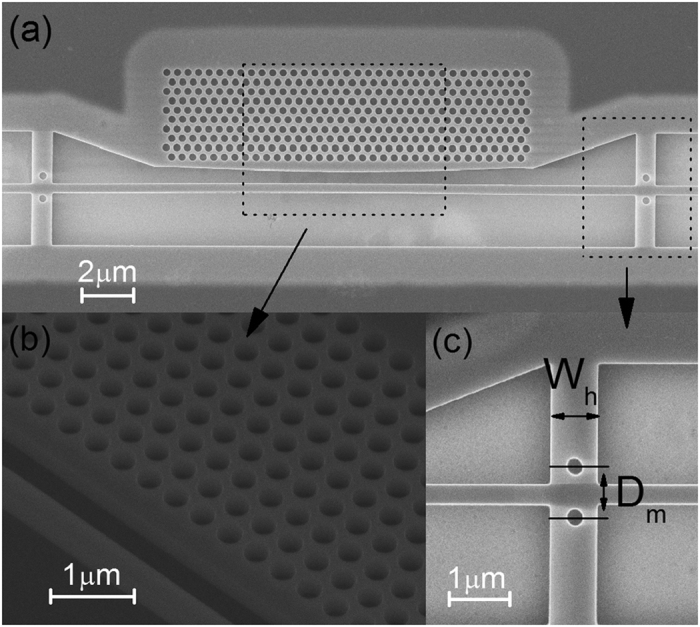
(**a**) The SEM image of the total fabricated side-coupled semi-2D-PhC cavity. (**b**) The local zoom-in SEM image of the cavity center. (**c**) The zoomed-in SEM image of the holder.

The overall scanning electron microscope (SEM) image of the fabricated side-coupled semi-2D-PhC cavity is shown in Fig. 5(a). From the enlarged view of the central part shown in Fig. 5(b), we can find that the size of the air holes have been fabricated with fairly good quality. In addition to the bus waveguide and PhC cavity, two holders for supporting the bus waveguide have been added to both sides of the cavity with a separation length of 20 μm. The details of these two holders are illustrated in Fig. 5(c), with a strip width *W*_*h*_ = 800 nm, hole radius of 150 nm and hole-hole distance *D*_*m*_ = *W*_*bus*_ + 500 nm. All these parameters have been optimized by FDTD simulations to ensure a high transmission of around 73% for double holders. From the measurement results, an insertion loss (IL) around 2–3 dB has been obtained for these holders, which is quite reasonable taking into account the fabrication errors. Although the holders introduced some loss, it significantly improved the mechanical stability of the bus waveguide during the undercutting process. Furthermore, the holders provide the bus waveguide with excellent tenacity and are suitable for optomechanical applications.

**Figure 6:**
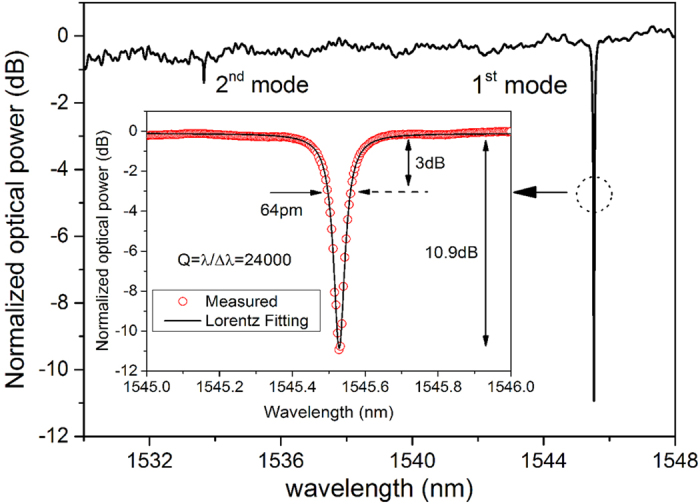
The normalized measured transmission spectrum of the side-coupled semi-2D-PhC cavity. The waveguide width and gap are chosen to be 375 nm and 500 nm, respectively. The inset is the zoomed-in transmission of the 1^st^ mode. Lorentz fitting is used for calculating the Q value.

We measured the transmission spectrum of the proposed device by scanning the tunable laser (Agilent 81600B) with a wavelength ranging from 1520 nm to 1610 nm, and the response of the device at the output port was detected by the power meter (Aglient 81635). The normalized transmission spectrum (normalized to that for a straight bus waveguide with the same width, also with a couple of holders with the same parameters) is shown in Fig. 6. The Lorentz fitting for the linear scale is also showed in the inset. It can be seen that the full-width half maximum (FWHM) of the peak is 64 pm, corresponding to a quality factor of *λ*/*∆λ* = 24000. The ER is around 10.9 dB, which is similar to the simulated result. The achieved Q is at least several times larger than that of the reported surface mode PhC cavity, and the ER value for our structure is also higher. In Fig. 6, we can see that the cavity demonstrated in our manuscript supports more than one mode within the bandgap, but these high order modes are well separated from the fundamental mode and the intensity of the high order modes are much smaller.

**Figure 7:**
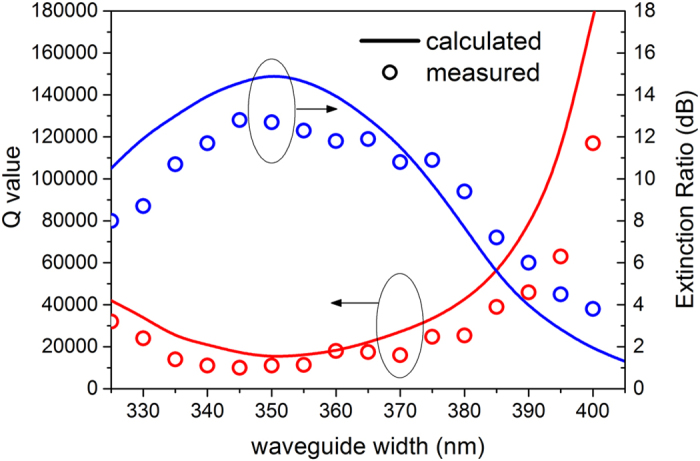
Measured and calculated Q values and ER of different waveguide widths with a fixed g = 500 nm.

To further study the performance of this cavity, especially of the intrinsic cavity, tuning of the mode matching is performed by adjusting the waveguide width while keeping other parameters fixed and *g *= 500 nm, which deviates from the condition of complete coupling. The calculated and experimental results are shown in Fig. 7, as a line and scatter, respectively. We know that there is a performance trade-off between the ER and Q value. The waveguide width we consider here is from 325 nm to 405 nm to ensure that the measured ER is higher than 3 dB. From Fig. 7, we can find that the measured results agree well with the calculated one. The deviation between the calculation and measurement is due to the fabrication errors, including the waveguide accuracy and surface roughness. The best complete-coupling region for a loaded cavity with a 500 nm gap is within a 340–360 nm waveguide width. Q (red curve) tended to increase and the ER (blue curve) tended to decrease as the waveguide width deviated from 350 nm. When the waveguide width is modified to 390 nm, a high Q of 46000 can be achieved, which is ~8 times larger than the reported surface-mode cavity with a Q of about 6200, while maintaining a similar ER (~6 dB) [20]. In particular, when the waveguide width is increased to 400 nm, an experimental Q up to 117000 can be obtained. In this case, the ER is dragged down to 3.8 dB. With further increases in width, the Q value gradually approaches the unloaded one (up to 10^6^) with an immeasurable ER. Since the gap between the bus waveguide and the PhC affects the coupling efficiency, we have also considered the cases *g *< 500 nm and *g *> 500 nm, which present similar tendencies on Q-ER trade-off, but the results are not shown here. In the case *g *< 400 nm Q is rather limited, though one can get a higher ER up to 30 dB which is qualified for “critical coupling”. ER is also insignificantly low in the case *g *> 500 nm. We thus suggest 400 nm* *< *g *< 500 nm to be a practical range with moderate Q-ER trade-off for side-coupled allocation and choose a gap width of 500 nm to achieve an acceptable extinction ratio with a rather high Q value in our work. This experimental high loaded Q cavity is nearly comparable to all-pass type ring resonators with similar size and permits single-mode operation [29], which enables multiple usages such as sensors and filters.

## Conclusion

In conclusion, we have demonstrated an FSR-free high-performance semi-2D-PhC cavity that synthesizes the advantages of both 2D and 1D photonic crystal cavities. A theoretical Q up to 6.7 × 10^7^ and mode volume V of 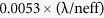
^3^ can be realized. An experimental Q value of 2.4 × 10^4^ and ER value of 10.9 dB with all-pass type output spectrum can be obtained simultaneously with a bus waveguide. A cavity with a larger Q up to 1.1 × 10^5^ can also be achieved by tuning the matching and coupling condition, while retaining an acceptable ER value. These unloaded or loaded (side-coupled) semi-2D-PhC cavities show great potential in applications such as sensors, filters, optomechanical devices and even lasers.

## Methods

### Simulation

FDTD method is used to calculate the band diagram, resonant mode and energy propagation of the PhC waveguide. Dipole source is chosen in the calculation of band diagram and unloaded Q, and mode source is used in the calculation of loaded Q with side-coupled bus waveguide. Perfectly matched layer (PML) boundary condition in y direction and periodic boundary condition at the interface of a unit cell in x direction are used for calculating the band diagram while PML outside the total cavity is used for Q calculation. Electric field-symmetric boundary condition is used in z direction for saving calculation time. Mesh step of 20 nm and 40 nm is set in the central part and outskirt of the cavity, respectively.

### Fabrication process

The fabrication of the proposed cavity was processed on a 220 nm-thick SOI wafer (SOITEC Inc.) with a buried oxide layer thickness of 2 μm. A positive tone electron beam resist PMMA (950K) with a thickness of 270 nm was used for masking, and E-beam lithography (Raith 150-II) was used for device patterning. The pattern was then transferred to the silicon layer through an inductively coupled plasma reactive-ion-etching (ICP-RIE) process with a gas mixture of SF6 and C4F8. A 70 nm shallow-etched grating coupler with a period of 630 nm and 50% duty cycle was fabricated at the end of the input/output bus waveguide to achieve efficient coupling to the single mode fibers (SMF). A BHF solution was then used for undercutting the buried oxide after the dry etching process and forming the suspended structures.

## Additional Information

**How to cite this article**: Zhang, J. *et al*. High-Q side-coupled semi-2D-photonic crystal cavity. *Sci. Rep.* **6**, 26038; doi: 10.1038/srep26038 (2016).
